# Perspectives on Tannins

**DOI:** 10.3390/biom11030442

**Published:** 2021-03-16

**Authors:** Andrzej Szczurek

**Affiliations:** Centre of New Technologies, University of Warsaw, S. Banacha 2C, 02097 Warsaw, Poland; a.szczurek@cent.uw.edu.pl

Tannins are a family of versatile, natural phenolic biomolecules whose main role is to protect plants against insects and fungi. Although this group encompasses a wide variety of oligomers and polymers, two main categories of tannins can be distinguished: hydrolysable tannins (HTs) and proanthocyanidins (Pas), known also as condensed flavonoid tannins, which are resistant to hydrolytic degradation [1]. Tannins contain aromatic rings bearing hydroxyl groups, which give them high chemical activity, causing them to form complexes with other macromolecules, such as carbohydrates [2], or bacterial cell membranes [3]. However, their main characteristics are complexation and precipitation of proteins [4]. Tannins exhibit high antioxidant properties applicable to the food and medical industries [5]. It has been shown that they prevent oxidative-stress-related diseases, such as cardiovascular disease, cancer, and osteoporosis. Due to their chemical reactivity, high availability in raw materials, and easy and safe extraction, tannins are widely used in the food, leather, and chemical industries [1]. Examples of current or potential applications include their use in the production of anticorrosive primers [6], as “green” alternatives to synthetic homologous phenolic compounds for the production of wood adhesives and particle boards [7], and in functional polymeric materials with variable physicochemical and morphological properties [8]. In addition, the pyrolysis of these materials, i.e., heat treatment in inert atmosphere at elevated temperatures, results in cellular carbonaceous materials [9], further broadening the range of possible applications. 

This book includes seven papers: five original articles and two review articles describing the versatile properties and applications of tannin biomolecules and tannin-based materials.

Sun et al. [10] investigated and described the role of epigallocatechin gallate (EGCg) in the conformation and agglomeration of human serum albumin (HSA). Their research was conducted in the presence or not of palmitic acid (PA). It was shown that EGCg increased the interdomain distance in HSA and HSA-PA. Regarding the effect of palmitic acid, the distance depended on the PA concentration. The EGCg also affected the secondary structure of the protein more significantly than palmitic acid. The EGCg decreased the α-helix content in a dose-dependent fashion. It was able to increase the HSA aggregation, whereas it promoted the formation of more heterogeneous aggregates. Any of these effects could impact the ability of serum albumin to transport and stabilize ligands, including EGCg and other polyphenols.

The article by Chen et al. [11] dealt with the applicability of microwave-assisted extraction to convert high molecular weight proanthocyanidins into monomeric catechins. The MSE extraction resulted in higher yields of monomeric catechins and PACs in comparison with conventional methods. Furthermore, the obtained extracts presented higher antioxidant capacity and α-glucosidase inhibitory activity than those prepared in conventional extractions. This finding suggests the potential use of the MAE products of grape seeds as functional food ingredients and nutraceuticals.

Guo et al. [12] revealed that proanthocyanins extracted from grape seeds might be able to serve as the effective dyes used to color cotton fabrics. They proved that proanthocyanins provided antibacterial, antioxidant, and UV protection to the treated cotton fabrics.

The study by Grobelna et al. [13] showed the higher stability and antioxidant activity of apple juice mixed with blue honeysuckle berry juice. The results were strictly due to the addition of the blue honeysuckle berry juice, which is rich in phenolic compounds (mostly anthocyanin).

Sepperer et al. [14] explored the capability of different types of tannins and tannin-based materials to capture ammonia. Industrial livestock farming produces enormous amounts of cattle manure, a source of ammonia. This is an important environmental issue due to the impacts on soil and air, and the European community has committed to reducing ammonia emissions by 30% as compared to 2005 by 2030. In their study, the ammonia adsorption capabilities of different types of tannins and tannin-based materials were considered. The obtained results were reasonably promising, with tannins showing a high ability to adsorb NH3 and reduce pH. These results open up new possibilities for the use of materials produced from tannins.

Braghiroli et al. [15] extensively reviewed the recent achievements related to the preparation of tannin-based gels and their carbon derivatives. In this review, all crucial aspects of gels preparation (synthesis, drying, and pyrolysis conditions) regarding their final properties were recalled and discussed. The review also provided examples of the practical application of received gels for energy storage, thermal insulation, and contaminant sorption in drinking water and wastewater.

Pizzi [16] reviewed known and established applications of tannin-based materials, as well as the future applications that are being developed at present and that promise to have an industrial impact in the future. The essential points of the materials, their drawbacks, and their likelihood of industrial application were briefly discussed. The chemical nature of these applications was described, for which it is essential to understand the roles of tannins and their derivatives.

This book, titled “Perspectives on Tannins”, presents articles and reviews disclosing the most relevant discoveries related to the antioxidant, antibacterial, and UV protection features of tannins. Polymerized tannin materials may find applications as adsorbents of toxic by-products. The chemical activity of tannins, their variety of forms, and their versatile properties make them suitable for a very wide range of applications.
